# Title-comparison of coronally advanced flap with chorion membrane vs coronally advanced flap with connective tissue graft in the treatment of multiple gingival recessions: a split-mouth randomised controlled study

**DOI:** 10.12688/f1000research.110829.1

**Published:** 2022-05-17

**Authors:** Sweta Pradhan, Neetha Shetty, Deepa Kamath

**Affiliations:** 1 Department of Periodontology, Manipal College of Dental Sciences, Mangalore, Manipal Academy of Higher Education, Manipal, Karnataka, 575001, India; 2 Professor and HOD, Department of Periodontology, Manipal College of Dental Sciences, Manipal Academy of Higher Education, Manipal, Karnataka, 575001, India; 3 Professor, Department of Periodontology, Manipal College of Dental Sciences, Manipal Academy of Higher Education, Manipal, Karnataka, 575001, India

**Keywords:** Chorion Membrane, Coronally advanced flap, Esthetics, Gingival recession, Perio-plastic surgery.

## Abstract

**Background:** The importance of esthetics has escalated over the years. The purpose of any perioplastic surgery is to address gingival recession while ensuring predictable root coverage and a pleasing appearance. An array of surgical procedures have been recommended for the management of recession defects. The present study compares the clinical and patient related outcome measures of coronally advanced flap with chorion membrane and connective tissue graft in the management of multiple adjacent gingival recessions.

**Methods:** The study was a prospective randomized controlled trial which included eight systemically healthy patients with an age range of 30-44 years with 36 labial/buccal, multiple adjacent, Cairo’s RT1 gingival recession defects, bilaterally.  CAF+CM was performed on one side whereas CAF+CTG was performed on the other side. The two groups were compared clinically at three and six months postoperatively.

**Results:** There was statistically significant decrease in recession depth, recession width, probing depth and clinical attachment level in both the groups from baseline to three and six months. However, intergroup comparisons revealed no statistically significant difference. At six months, both groups showed statistically significant improvements in keratinized tissue width and gingival thickness. The gingival thickness of the CAF+CM group increased significantly at three and six months. In terms of root coverage aesthetic score (RES), there was no significant difference observed between the two groups. In terms of patient reported outcome measures (PROMS), patients preferred the CAF+CM technique.

**Conclusion:** Within the limits of the current study, the use of chorion membrane resulted in considerable root coverage and increased gingival thickness. Periodontal regeneration can be facilitated by the distinctive features of the chorion membrane. Coronally advanced flap plus chorion membrane is a novel approach for root coverage procedures.

## Introduction

An attractive smile contributes significantly to esthetics as well as self-confidence and health. An ideal smile should be determined by the anatomical relationship between the teeth, the periodontium, and the surrounding oral tissues. When any of these components are out of harmony, the result is a smile that is perceived as unattractive. ^1^ Periodontal disease alters the relationship between teeth and gingiva. Treatment for periodontal disease for centuries has been focused more on preserving and restoring periodontal health than achieving aesthetically pleasing result. ^2^

Gingival recession describes the migration of the marginal gingiva beyond cementoenamel junction. ^3^^,^^4^ Aesthetic considerations, dentinal hypersensitivity, root caries prevention, and cervical abrasions are the primary indications for root coverage procedures. ^5^ This condition often negatively impacts aesthetics when it occurs in the anterior regions. Therefore, many patients seek cosmetic correction in order to meet their aesthetic and functional demands, which remains a major therapeutic challenge. ^6^^,^^7^ The coronally advanced flap, double papilla rotating flap, laterally moved flap, subepithelial connective tissue graft, free gingival graft and their variants were proposed for the management of recession in the last 60 years. ^7^^,^^8^ Some authors have coupled allografts such as acellular dermal matrix, ^9^ collagen matrix, ^10^ and platelet concentrates such as Platelet rich fibrin and plasma ^11^ with some of the above stated procedures, particularly the coronally advanced flap approach. Every technique has its own indications, contraindications, advantages, and disadvantages. The management of recession defects has been the subject of numerous systematic reviews and meta-analyses. Coronally advanced flap with connective tissue grafts have been proved to be the gold standard for addressing multiple gingival recession defects in many studies. ^12^^,^^13^ But this approach is associated with several major drawbacks such as: the addition of another surgical site to obtain an autologous soft tissue graft, presence of vertical incisions, which can create discomfort and delayed healing for patients.

Recent literature documents the use of newer materials like the Placental Membranes such as the chorion membrane. ^14^ Chorion membranes are placental allografts that emerge as a versatile and novel material. These allografts hold antimicrobial and antibacterial properties and are immunomodulatory. They have special biological features that aid wound healing and regeneration. ^15^^,^^16^ According to studies, fresh chorion has a higher load of growth factors and cytokines. ^15^ Since decades, placental membranes have been used for various medical purposes. Currently, these membranes are used in root coverage procedures.

The reticular layer, basement membrane, and trophoblast layer are the three layers of the chorion membrane. Collagen fibres, fibronectin, proteoglycans, and laminin make up the extracellular matrix of the chorion membrane. Collagen Types I, III, IV,V, VI are present and are well tolerated with inherent haemostatic properties, as well as being bioabsorbable, allowing epithelial cells and surrounding autogenous connective tissue to migrate. ^17^^,^^18^ Fibronectin is vital for tissue repair, blood coagulation, cell migration, and adhesion. ^19^ Cell proliferation, cell-to-cell adhesion, cell expansion, and cell differentiation is promoted by laminin. ^20^

In light of these biologic properties, we hypothesized that coronally advanced flap with Chorion membrane would be effective in management of multiple gingival recession defects.

The aim of the study is to compare and evaluate the clinical and patient related outcomes of coronally advanced flap plus chorion membrane and coronally advanced flap plus connective tissue graft in the management of multiple gingival recession defects.

## Methods

### Study design

The clinical and patient-related outcome measures of coronally advanced flap utilising chorion membrane (CAF+CM) as the test site and coronally advanced flap using connective tissue graft as the control site (CAF+CTG) are compared in this prospective, randomised, comparative split mouth study. Over the course of six months, this clinical trial was conducted. This study protocol was approved by the Research Ethics Committee of MCODS, Mangalore (Ref no-19088) and registered with the Clinical Trial Registry of India (CTRI No- 039332). Before enrolment, all participants signed written informed consent forms. All procedures were conducted in compliance with the Helsinki Declaration 1975.

*Study subjects*

Eight study participants were chosen from the outpatient department of Periodontics at Manipal College of Dental Sciences Mangalore, MAHE University, Karnataka, India.

*Sample size calculation*

$$N=2{\left(Z_{1-\propto /2}+Z_{1-\beta }\right)}^{2}\sigma^{2}/d^{2}$$

Using the above formula, the sample size was calculated.

Where Z (1-α/2) = Z score for the α error chosen.

Z (1-β) = Z score for the power chosen.

σ = average standard deviation. d = the minimum difference between in the values with which make clinically relevant impact.

Based on the study by Lafzi *et al*, ^21^ the study contained an eight-patient sample size with 80 percent power and a clinically significant difference of 0.9 units.

*Randomization*

The investigator (DK) was in charge of enrolling participants and allocating surgical procedures. The coin toss approach was used for randomization. The lead investigator (SP) performed all surgical operations. A masked investigator (NS) assessed the patients during the recall period and was blinded to all the surgical treatments that had been assigned.

### Inclusion criteria for the patients

1. Patients who had signed the informed consent and were willing to be a part of the study.
2. Patients with age range of 20-50 years.
3. Patients fulfilling Cairo classification ^22^ of Recession Type 1 (RT1) involving any two teeth on either side of the midline extending from central incisors to the premolars in either maxilla or mandible.
4. Full mouth Plaque score less than one
5. Full mouth Mombelli’s sulcular bleeding score less than 20%
6. Cervical abrasion with or without restorations.

### Patients those were excluded from the study were with

1. Compromised systemic illness
2. Pregnancy
3. History of root coverage
4. Need for antibiotic prophylaxis
5. Patients with habit of smoking.
6. Teeth with buccal and lingual inclinations.
7. Patients with fixed orthodontic appliances.

### Interventions

Eight patients had been assessed for eligibility and allocated to interventions. As it is a split mouth study, both the interventions were done on the same patient. On one side, CAF+CHORION MEMBRANE (Test Group) was done whereas CAF+CTG (control group) was done on the other side. After 4 weeks of the first intervention on one side, patient was recalled for the second intervention on the other side.

Clinical measurements were taken at baseline, three and six months after surgery. The measurements were

1. *Plaque Index by Sillness and Loe (1964)* ^23^
2. *Modified sulcular bleeding index by Mombelli et al (1987)* ^24^
3. Recession Depth (RD)
4. Recession Width (RW)
5. Probing Depth (PD)
6. Clinical attachment level (CAL)
7. Width of the Keratinized Tissue (WKT)
8. Gingival Thickness (GT)
9. Root coverage esthetics score (RES)
  Mean and complete root coverage percentages were determined after six months.
10. Patient Reported Outcome Measures (PROMS)

**Figure 1.**
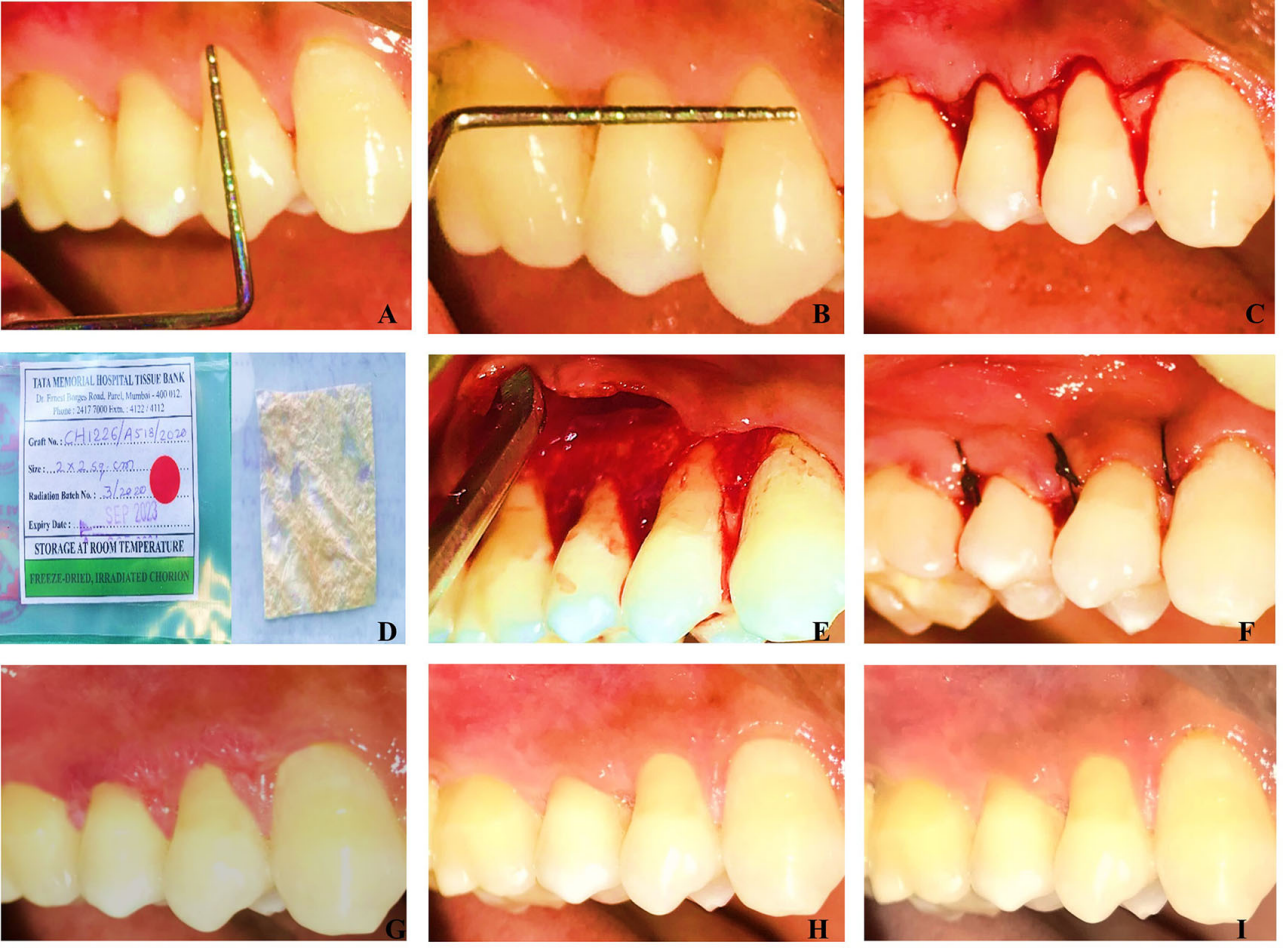
A&B: Pre-operative recession depth and width measurement of the CAF+CM Group. C: Horizontal Incisions were made and Split-full-split thickness flap reflected. D: Freeze dried irradiated chorion membrane from Tata Memorial Hospital Tissue Bank. E: Placement of Chorion membrane at the defect site. F: Independent Sling sutures placed. G: 15 days post-operative view of the site. H: Three months post-operative view of the site treated by CAF+CM. I: Six months post-operative view of the site treated by CAF+CM.

**Figure 2.**
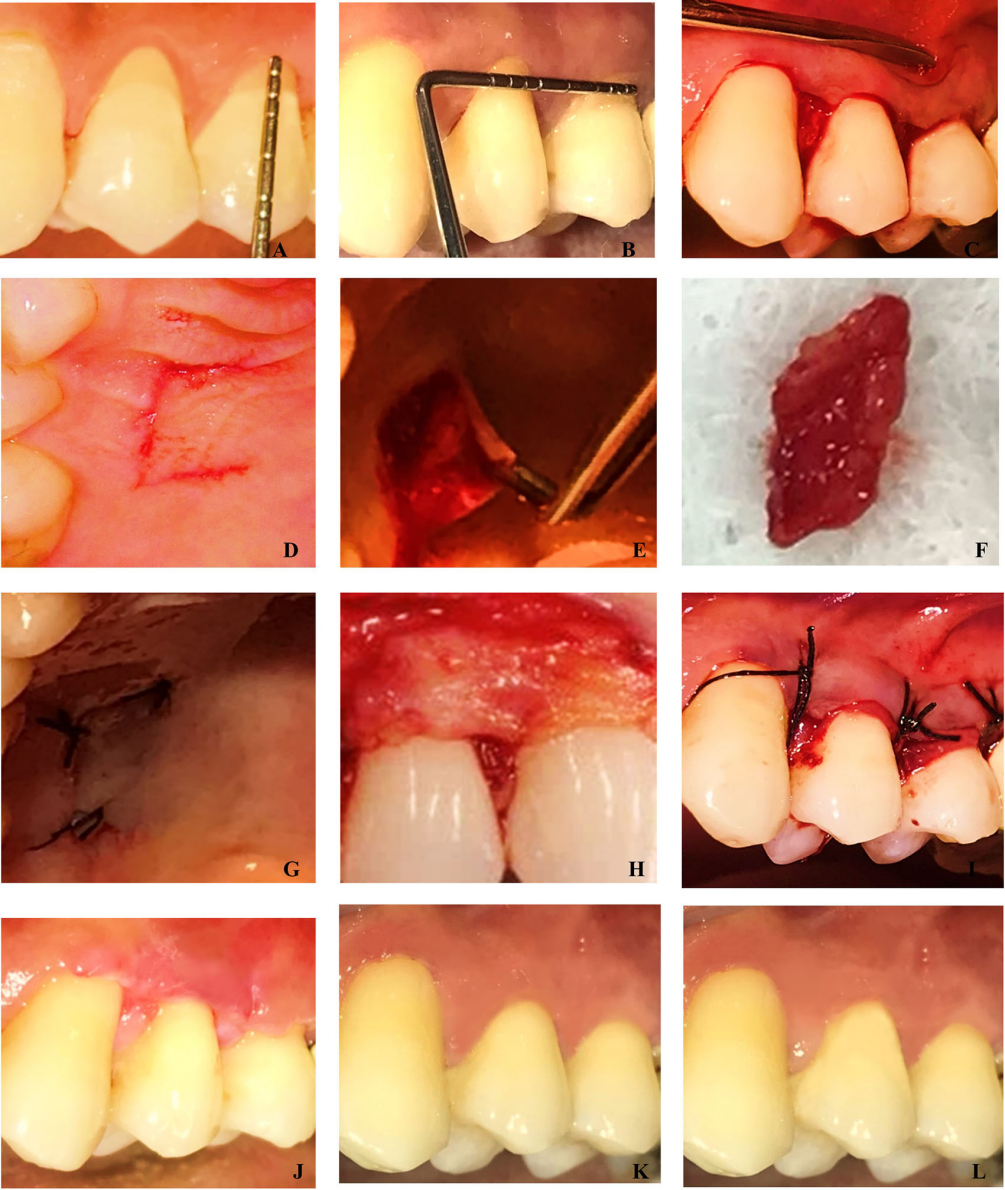
A&B: Pre-operative recession depth and width measurement of the CAF+CTG Group. C: Split-full-split thickness flap reflected. D: Trap door incision for harvesting CTG E: Harvesting of the CTG. F: Connective tissue graft. G: Suturing at the donor site H: CTG placed on the defect site. I: Flap coronally positioned and sutured with independent sling sutures. J: 15 days post-operative view of the site. K: 3 Months post-operative view. L: Six months post-operative view of the site treated by CAF+CTG.

The baseline pre-operative Clinical measurements were shown in Figures 1A, 1B and 2A, 2B.

### Pre surgical procedure

For all the patients, a complete medical and dental history, periodontal assessment using clinical parameters, radiographs, and clinical pictures were taken. Initial treatment included instructions for oral hygiene maintenance and full mouth prophylaxis. Buccal prominences, as well as trauma from occlusion, were treated with coronoplasty. To curtail tooth brushing trauma, patients with recession type defects were advised to use a modified Stillman Brushing technique. All research participants were advised to rinse for one minute with 0.2 percent Chlorhexidine gluconate, prior to the surgery. The surgical procedure was done under complete asepsis and infection control protocols. The baseline clinical pictures are presented in Figures 1 (A, B) and 2 (A, B).

**Surgical preparation**

The surgical technique adopted in this study was the envelope coronally advanced flap put forward by Zucchelli and De Sanctis (2000). ^25^

Local Anaesthesia (2% lidocaine with 1:80000 epinephrine) was administered. The exposed root surfaces were planned with curettes. This was done to eliminate any debris, calculus, soft tooth structures or undercuts. The root surfaces were irrigated with saline solution after instrumentation to remove any detached fragments from the defect site and operative field. Horizontal incisions at the level of the cementoenamel junction (CEJ) were made, followed by sulcular incisions in the buccal aspect of the affected teeth with a No 15 BP blade. The split-full-split flap technique was performed. The papilla was deepithelialized in the interdental area. The flap was reflected in full thickness apical to the gingival margin to allow the periosteum to cover the avascular root surfaces. To assist the coronal repositioning of the flap, it was elevated partially beyond the mucogingival junction ( Figures 1C, 2C).

**Test group (CAF+CM)**

Approximately five to ten minutes prior to surgery, the chorion membrane was removed from the cryo-box container according to the manufacturer’s protocol. The cryopreserved chorion membrane was cut according to the recipient site size before being transplanted as shown is Figure 1 (D, E).

**Control group (CAF+CTG)**

***Harvesting of the connective tissue graft*** ^26^

Palatal infiltration was administered with 2% lidocaine with 1:80000 epinephrine. In the palate, a horizontal partial thickness incision 3-5mm apical to gingival margin was performed. Vertical releasing incisions were made mesiodistally, roughly corresponding to the length and width of the required graft. An initial partial thickness flap was raised parallel to the palatal gingiva in the centre of the palate. The connective tissue graft underneath was visible. The blade contacted the bone during the secondary incision. The connective tissue graft was reflected with a tiny periosteal elevator or Kirkland knife. After harvesting the connective tissue graft, interrupted sutures were used to approximate the flap ( Figure 2D, E).

***Placement of CTG***

A no.15 BP blade was used to trim CTG according to the required size. In the recipient site, after reflecting the split-full-split flap, CTG was placed at the defect area and stabilised as shown in Figure 2F.

The reflected flap was advanced coronally and sutured over the papilla that had been de-epithelialized. Sling sutures with 5-0 non-resorbable sutures were used to stabilise the flap without causing any tension as presented in Figure 1F and Figure 2I. At the defect site, a periodontal pack was applied.

### Post-surgical care

To avoid post-surgical infections, all patients were given Cap Amoxicillin 500 mg thrice daily and Tab Lyser-D twice daily for 5 days. For two weeks, all the patients were told to use 10 mL Chlorhexidine Mouth Rinse (0.2%) twice a day. All the patients were instructed to maintain good oral hygiene and abstained from brushing their teeth in the operative field for a period of 14 days. Two weeks following surgery, the sutures and periodontal pack were removed. All the patients were recalled after 7, 14, 25, and then once a month for the next six months. De-plaquing was carried out for all the patients at follow up visits.

The post-operative clinical pictures of = were presented in Figures 1 (G, H, I) and 2 (J, K, L).

### Statistical analysis

Statistical analysis was implemented on the data gathered at baseline, three and six months. Recession depth (RD), Recession width (RW), and Clinical Attachment Level (CAL) were the primary outcome variables, while the rest were secondary. SPSS Version 20.0, a commercially accessible programme, was used to conduct the statistical analysis. For each parameter, the mean+SD for the clinical variables was determined. For intragroup comparisons, the paired t-test was utilised. Independent t-test was applied for intergroup comparisons.

## Results

Eight systemically healthy patients with labial/buccal, multiple adjacent, maxillary, or mandibular Cairo’s RT1 gingival recession defects, bilaterally, with a mean age of 35.88 year (mean age range: 30-44 years) were assessed and randomly assigned as shown in Table 1. There were 36 recession defects addressed in total, with 18 defects each in both test and control group. No statistically significant differences were observed between the CAF+CM group and CAF+CTG group in presurgical/baseline parameters.

**Table 1.** Total number of patients.

Gender	Frequency	Percent
Female	4	50.0
Male	4	50.0
TOTAL	8	100

There were no dropouts in the study. No adverse effects were observed for the duration of the study. All patients experienced uneventful healing.

At baseline, three and six months, the patient’s mean PI were 0.86±0.05, 0.75±0.12, 0.83±0.05 respectively. The patient’s mean mBI were 19.3±0.75, 18.65±0.97 and 18.85±0.88 at baseline, three months, and six months respectively. As indicated in Table 2, plaque and bleeding scores decreased from baseline to three months and six months.

**Table 2.** Intra-group comparison of Plaque and sulcular bleeding Indices at baseline, three and six months.

	N	Mean ± SD			Mean difference ± SD			Intragroup comparisons (P Value)		
		Baseline (BL)	3 months	6 months	BL-3 months	BL-6 months	3 months-6 months	BL-3	BL-6	3-6 months
**Plaque index**	08	0.86±0.05	0.75±0.1	0.83±0.05	0.11±0.1	0.04±0.07	0.08±0.09	0.051	0.197	0.048
**Bleeding index**	08	19.3±0.75	18.65±0.9	18.85±0.8	0.65±0.6	0.45±0.51	0.2±0.26	0.023	0.042	0.064

The baseline RD in the CAF+CM group was 2.72±0.67. At three months, the recession depth reduced to 0.72±0.73 and at six months, it was 0.78±0.73. In the other group i.e., CAF+CTG, the mean values at baseline, three and six months were 2.67±0.84, 0.44±0.62 and 0.5±0.62 respectively. The RW at baseline, three and six months in the CAF+CM group were 3.11±0.76, 1.39±1.24 and 1.44±1.25 respectively. In CAF+CTG group, the RW at baseline was 3.17±0.51. The RW reduced to 0.61±0.923 at three months and 0.72±0.96 at six months. The Probing depth at baseline was 2.72±0.46 in CAF+CM group. At three months and six months, it reduced to 2.25±0.49 and 2.33±0.49 respectively. In the CAF+CTG group, the baseline PD was 2.67±0.49 and at three and six months was 2.33±0.49. The CAF+CM group showed mean CAL of 5±0 at baseline, 3.03±0.87 and 3.11±0.83 at three and six months. CAF+CTG group exhibited CAL of 5.33±1.08 at baseline and at three and six months, 2.78±0.94 and 2.72±0.89 respectively. The mean WKT in the chorion membrane group at baseline was 3.18± 0.64 and at three and six months, the mean WKT increased to 4.35±0.49. In the connective tissue graft group, the WKT was 3.17±0.86 at baseline and at three and six months, the WKT was 4.17±0.86. The Gingival thickness (GT) was 1.56±0.57 at baseline in the CAF+CM group. There was significant increase of 1.56 at six months while in CAF+CTG, the baseline GT was 1.53±0.44. At three months, it was 2.5±0.51. There was increase of 0.006 ±0.16 at six months. The root coverage percentage achieved at the end of six months in the chorion membrane plus CAF group was 73.7% and, in CAF + CTG group was 84.1%. The root coverage esthetics score at six months for the chorion membrane group plus CAF was 8.5±1.6 and for CTG group was 9.25±1.39. Tables 3, 4 and 5 indicate the mean changes in the study variables at all the three time points.

**Table 3.** Baseline characteristics of test and control groups.

Parameters at baseline	Groups	N	Mean ± SD	Intergroup comparisons difference	P value
RD-BL	Test Group	08	2.72±0.67	0.06±0.87	0.79
	Control Group		2.67±0.84		
RW-BL	Test Group	08	3.11±0.76	0.06±0.73	0.749
	Control Group		3.17±0.51		
PD-BL	Test Group	08	2.72±0.46	0.06±0.73	0.749
	Control Group		2.67±0.49		
CAL-BL	Test Group	08	5±0	0.33±1.08	0.21
	Control Group		5.33±1.08		
WKT-BL	Test Group	08	3.18±0.64	0.06±0.66	0.718
	Control Group		3.17±0.86		
GT-BL	Test Group	08	1.56±0.57	0.03±0.5	0.816
	Control Group		1.53±0.44		

RD=Recession depth, RW=Recession width, PD=Probing Depth, CAL=Clinical Attachment level, WKT=Width of keartinised tissue, GT=Gingival thickness.

**Table 4.** Clinical outcomes at 3 months.

Parameters at 3 months	Groups	N	Mean ± SD	Intergroup comparisons difference	P value
RD-3	Test Group	08	0.72±0.73	0.28±0.77	0.145
	Control Group		0.44±0.62		
RW-3	Test Group	08	1.39±1.24	0.78±1.4	0.03 *
	Control Group		0.61±0.92		
PD-3	Test Group	08	2.25±0.49	0.08±0.73	0.636
	Control Group		2.33±0.49		
CAL-3	Test Group	08	3.03±0.87	0.25±1.1	0.349
	Control Group		2.78±0.94		
WKT-3	Test Group	08	4.35±0.4	0.22±0.7	0.215
	Control Group		4.17±0.8		
GT-3	Test Group	08	3.11±0.68	0.61±0.5	<0.001 *
	Control Group		2.5±0.51		

RD=Recession depth, RW=Recession width, PD=Probing Depth, CAL=Clinical Attachment level, WKT=Width of keratinized tissue, GT=Gingival thickness.

* p=0.05.

**Table 5.** Clinical outcomes at six months.

Parameters at 6 months	Groups	N	Mean ± SD	Intergroup comparisons difference	P value
RD-6	Test Group	08	0.78±0.73	0.39±1.04	0.13
	Control Group		0.5±0.62		
RW-6	Test Group	08	1.44±1.25	0.72±1.27	0.028 *
	Control Group		0.72±0.96		
PD-6	Test Group	08	2.33±0.49	0±0.77	1
	Control Group		2.33±0.49		
CAL-6	Test Group	08	3.11±0.83	0.58±1.18	0.051
	Control Group		2.72±0.89		
WKT-6	Test Group	08	4.35±0.4	0.22±0.7	0.215
	Control Group		4.17±0.8		
GT-6	Test Group	08	3.11±0.68	0.56±0.59	<0.001 *
	Control Group		2.56±0.48		

RD=Recession depth, RW=Recession width, PD=Probing Depth, CAL=Clinical Attachment level, WKT=Width of keratinized tissue, GT=Gingival thickness.

* p=0.05.

## Discussion

This is the only study that we are aware of that compares both clinical and patient-related outcome measures of coronally advanced flap with connective tissue graft and chorion membrane. In the present study, we used the Zucchelli’s (2000) technique of CAF, ^25^ i.e., without vertical incisions to get good aesthetical outcomes. Hofmanner *et al* ^27^ published a systematic evaluation on predictability of root coverage procedures. The root coverage acquired with CAF without vertical releasing incisions was found to be steady over a five-year period, according to the systematic review’s findings. Graziani *et al.* ^28^ published a comprehensive review and meta- analysis, stated that there is no “one perfect surgical method” for treating multiple gingival recession defects. The evidence states that usage of a graft or modification of flap design, may improve the clinical results of root coverage. Therefore, a plethora of regenerative procedures were combined with coronally positioned flap techniques to enhance the predictability.

According to the recent literature, the gold standard technique is coronally advanced flap with subepithelial connective tissue graft. ^12^ The drawback with this is the procurement of CTG which requires an additional surgical site and many a times, there is limited availability of the graft which hinders the treatment. To bypass the need of another surgical site and ensure sufficient graft availability and increase patient acceptance, a wide array of graft biomaterials such as chorion membranes have been described in the literature, as a suitable alternative to CTG. There are very few studies that have assessed the efficacy of chorion membrane for root coverage. The direct comparison between Zucchelli’s technique ^25^ of coronally positioned flap using chorion membrane or connective tissue graft have been subjected to limited investigation. There are inconsistent data available based on the esthetic and the patient related outcome measure, therefore further investigations comparing these two techniques are needed.

Thus, the split mouth randomized clinical study consisting of eight systemically healthy patients was attempted to compare and evaluate the clinical and patient related outcome measures of coronally advanced flap without vertical releasing incisions (CAF) by using CTG and/or CM in the management of Cairo’s RT1 multiple gingival recessions. The present study was accomplished in six months. There were no significant difference observed in terms of the clinical parameters at baseline between the two groups. The results revealed that both biomaterials were extremely effective at obtaining root coverage.

The mean Plaque Index (PI) ^23^ and Mombelli’s Sulcular Bleeding Index (BI) ^24^ scores were < 1 and <20% respectively throughout the study period. This is because the patient maintained their oral hygiene throughout the study period along with periodic recall for professional oral prophylaxis at one, three and six months. From baseline to three and six months, there was a decrease in the Plaque and Bleeding Index. These findings can be substantiated by the fact that since the patients included in this study had a good degree of oral hygiene but with gingival recession, implying that these patients used to employ incorrect brushing techniques. This is in agreement with the findings of Tezel ^29^ and Oliveira *et al* ^30^ who stated that faulty tooth brushing with medium bristle toothbrush leads to periodontal attachment loss and eventually leads to gingival recession.

There were insignificant differences among the two groups when comparing the mean values of recession depth at all the three time points. When the disparities between both the groups are compared from baseline to three months and three-six months, the control group’s mean value is higher by 0.22, although the difference is statistically insignificant (p value=0.227). In a study by Lafzi *et al*, ^21^ which is similar to the current investigation, the values from baseline to three months revealed substantial results. However, in our trial, the baseline RD was lower compared to the previous study. According to Huang *et al* ^31^ variations in recession depth following a coronally positioned flap procedure are linked to the baseline depth of recession defect. At baseline, the mean recession width in the CAF+CM group was 3.11±0.76 with mean recession widths of 1.39±1.24 and 1.44±1.25 at three and six months respectively. These findings are consistent with Sharma *et al* ^32^ study, in which the author analysed for six, 12, and 36 months and found a significant difference. Other studies previously mentioned such as in Chakraborty *et al* study ^33^ reported similar findings. The differences among the groups in terms of probing depth at baseline, three and six months were found to be statistically insignificant. The current study’s findings were similar to those of Gupta *et al* study. ^14^ In both the groups, the clinical attachment level increased approximately by two mm. No significant variations in CAL were identified between both the groups at three and six months. The gain in CAL can be interpreted as some form of root surface attachment. On comparing the mean width of the keratinised tissue (WKT) at three and six months, the mean WKT of the CAF+CM was higher with a difference of 0.22 but was statistically insignificant. The difference in keratinized tissue width observed by histochemical and visual methods at 3 and 6 months, according to Lafzi *et al* ^21^ study were also found to be insignificant. When the mean gingival thickness (GT) of both the groups were compared at three and six months, the CAF+CM group’s mean GT was greater, with a difference of 0.61 and 0.55 respectively and the findings were significant statistically when compared with the baseline values. These findings are supported by several studies and case studies. Ghahroudi *et al.* (2013) ^34^ in a study and Aravind S (2015) ^35^ in a case report have observed increase in width of the keratinized tissue and thickness with amniotic membrane when compared to CTG. It might be because of the presence of keratinocyte growth factor which is released from the amnion-chorion membrane and that helps the mucogingival junction retain its position by promoting keratinization of the epithelial cells. ^34^ The percentage of root coverage attained at six months was 73.7% and 84.1% in CAF+CM and CAF+CTG groups respectively. The connective tissue group revealed slightly more percentage of root coverage than the chorion membrane group, but the difference was statistically not significant. The Root coverage esthetic score (RES) in the present study for CAF+CM group was 8.5±1.6 and for CAF+CTG group was 9.25±1.39. No significant differences on mean RES were observed on comparing both the groups (Pvalue-0.17). The high esthetic scores in both the groups can be attributed to several factors. First, there were no releasing incisions given, which preserves the blood supply of the flap. The preservation of papillary integrity aids in more aesthetically pleasing wound healing. Second, using chorion membrane and connective tissue grafts allows for better colour and texture matching with the surrounding area. Third, using microsurgical instruments allows for precise manipulation of soft tissue and suturing, which improves primary wound closure and stability.

Patient reported outcome measures (PROMS) were evaluated in this study using a questionnaire and visual analogue scale to evaluate dentinal hypersensitivity, esthetic outcomes, and other comorbidities such as post-operative pain, bleeding etc. PROMs were included as one of the defining parameters in our study, which is in line with the recent consensus and systematic review published by American Academy of Periodontology and Cairo. ^36^^,^^37^ The results obtained reveal considerable difference between the two treatment modalities, with coronally advanced flap plus chorion membrane being the favoured surgical procedure among the patients. This is mostly owing to the creation of another surgical site in the connective tissue group, which in some cases resulted in post-operative pain. There were no adverse reactions during the study. The chorion membrane’s self-adhesiveness is a bonus feature. This biologic membrane binds to the tissues when it comes into contact with them, eliminating the need for additional suturing and simplifying the treatment procedure. In our study, with the use of Chorion membrane with CAF elevated the patient satisfaction with a good color match, soft tissue texture and root coverage.

The present study has a few limitations. The sample size taken was small and the follow-up period was also short. Short-term analyses, such as the one used in this study, cannot determine the stability of root coverage. However, more studies on histological evaluation of chorion membrane should be focussed to assess regeneration of the periodontal tissues.

## Conclusions

The following conclusions were drawn from analysis of the results and limitations of the present study:

• In this study, both treatment groups demonstrated significant improvements in root coverage. When comparing the gold standard method CTG and Chorion membrane with coronally advanced flap, no significant differences were observed. Hence, supporting the use of Chorion membrane for the management of multiple adjacent recession defects.
• When compared to CAF+CTG group, the CAF + Chorion membrane demonstrated a substantial increase in gingival thickness. This is mostly due to the chorion membrane’s distinctive characteristics and its delayed resorption rate. It can hold its physical form up to 4 weeks. This ensures that the chorion membrane leads to periodontal regeneration if they can retain their form till 4 weeks.

### Future directions

• Further studies should be done in future to explore the effectiveness of Chorion membrane in root coverage procedures with the help of surgical microscope. This may result in complete root coverage due to enhanced precision and manipulation of the tissues under magnification.
• Computerized image analysis should also be explored to better understand the clinical outcomes of root coverage.

## Data availability

### Underlying data

Figshare: Underlying data for “Comparison of coronally advanced flap with chorion membrane vs coronally advanced flap with connective tissue graft in the treatment of multiple gingival recessions: A split mouth study”. DOI: https://doi.org/10.6084/m9.figshare.19401410.v5 ^38^

This project contains the underlying following data:

• Data file 1: Master Chart for both the group.xlsx (Table containing the raw data of the study)
• Data file 2: Master chart for Plaque and Bleeding Index.xlsx
• Data file 3: Demographic data.xlsx
• Data file 4: Percentage of root coverage calculation.xlsx
• Data file 5: Sample size calculation formula
• Data file 6: Statistical analysis

Data are available under the terms of the Creative Commons Attribution 4.0 International license (CC-BY 4.0).

### Extended data

Figshare: Extended data for “Comparison of coronally advanced flap with chorion membrane vs coronally advanced flap with connective tissue graft in the treatment of multiple gingival recessions: A split mouth study”. DOI: https://doi.org/10.6084/m9.figshare.19402178.v3 ^39^

• This project contains the following data
• Case Performa
• Questionnaire
• Informed consent form in three languages (English, Kannada and Malayalam)
• Ethical Committee Approval letter
• Clinical Trial Protocol

Data are available under the terms of the Creative Commons Attribution 4.0 International license (CC-BY 4.0).

## Reporting guidelines

Figshare: CONSORT check list and flow chart for “Comparison of coronally advanced flap with chorion membrane vs coronally advanced flap with connective tissue graft in the treatment of multiple gingival recessions: A split mouth study”. DOI: https://doi.org/10.6084/m9.figshare.19401587.v3 ^40^

Data are available under the terms of the Creative Commons Attribution 4.0 International license (CC-BY 4.0).

## Authors’ contribution

All the authors have equal contribution to this research in manuscript preparation, data collection and interpretation.

## Competing interests

No competing interests were disclosed.

## Grant information

The author(s) declared that no grants were involved in supporting this work.
