# Supplementary material for: Title-comparison of coronally advanced flap with chorion membrane vs coronally advanced flap with connective tissue graft in the treatment of multiple gingival recessions: a split-mouth randomised controlled study
Source: F1000Res. 2022 May 17;11:533. [Version 1] doi: 10.12688/f1000research.110829.1 (PMC9171291; doi:10.12688/f1000research.110829.1)
Supplement: Peer review history

# Peer review: Reviewer response for version 1

**DOI:** 10.5256/f1000research.122480.r138134

**Published:** 2022-06-06

**Authors:** Neelesh Singh

**Affiliations:** 1 Department of Periodontology, Babu Banarasi Das College of Dental Sciences, Babu Banarasi Das University, Lucknow, Uttar Pradesh, India

1. Title of the study is well going with the work and self explanatory.
2. The study is an excellent attempt towards research on the management of Gingival Recession. It is a comparison of Chorion membrane vs CTG using a similar flap “Coronally Advanced Flap”. It's an attempt to achieve success with a material that is readily available so that patient’s treatment can be achieved and even preventing the secondary trauma to the palate for the graft (CTG) procurement.
3. Its is an interesting study and the authors have collected a unique dataset using cutting edge methodology. The paper as a whole is generally well written, structured and concluded.
4. The Introduction provides an overview of the history of the two techniques in question and the need of present study followed by a robust explanation of the materials and methods including stats.
5. All the cases present depict the results clearly. The power of study is good and the advantageous thing is that it is a split mouth pattern. Furthermore, the pictures taken are clear. The results are well jotted and easy to understand for the reader.
6. The main part 'Discussion' is extremely well structured with emphasis on the deficiencies and comparison to the previous classic research on the related techniques. The authors have even explained the short coming, which is appreciable.  

The paper as a whole marks a good step in periodontal research and can be Approved for indexing.

Is the work clearly and accurately presented and does it cite the current literature?

Yes

If applicable, is the statistical analysis and its interpretation appropriate?

Yes

Are all the source data underlying the results available to ensure full reproducibility?

Yes

Is the study design appropriate and is the work technically sound?

Yes

Are the conclusions drawn adequately supported by the results?

Yes

Are sufficient details of methods and analysis provided to allow replication by others?

Yes

Reviewer Expertise:

Periodontologist

I confirm that I have read this submission and believe that I have an appropriate level of expertise to confirm that it is of an acceptable scientific standard.

# Peer review: Reviewer response for version 1

**DOI:** 10.5256/f1000research.122480.r138132

**Published:** 2022-05-24

**Authors:** Dr. Preetha Jayasheela Shetty

**Affiliations:** 1 Department of Biomedical Sciences, College of Medicine, Gulf Medical University, Ajman, United Arab Emirates

The title of the article, **“**comparison of coronally advanced flap with chorion membrane vs coronally advanced flap with connective tissue graft in the treatment of multiple gingival recessions: a split mouth study”, accurately, succinctly, and appropriately expresses the theme and contents of the study.

 The introduction gives a comprehensive overview of the management of gingival recession defects, identifies the drawbacks associated, and summarises the current scenario. Chorion membrane is a very novel material that has been used in the medical front for many years. It is very recently that this versatile membrane has been used in dentistry. The authors have done justice in citing the novelty of the membrane and the superiority of the material in the field of periodontics. The authors have clearly mentioned the purpose of the study, which was to compare the two materials, namely Chorion membrane and connective tissue graft, in the management of gingival recession defects. The research question thus proposed has been substantiated with proper evidence.

 The author has succeeded in clearly describing the necessary armamentarium required for the research and has also chalked down a step wise methodology, which would be of great help to even other fellow researchers, who wish to carry out future research on this novel technique. A randomised clinical trial is evaluated by how comprehensibly the randomisation process has been described and followed. The authors have done justice to this aspect by outlining the randomisation process in a very systematic manner. The split mouth design of the study has also been justified. Thus, the selection of the study design is justified and the work showcases academic merit.

 Student’s t test was done for the intra and inter group comparisons which appropriately provides statistical support to the data. The statistical analysis and its interpretation look appropriate.

 The results and interpretations are sound and supported by relevant and current evidence. The authors have stated the findings, highlighting key aspects in tables and figures. The results with significant and non-significant values also have been cited well in the study. All the source data, underlying the results, are available to ensure a high level of reproducibility.

 The study provides initial evidence towards the advantage of the chorion membrane substantiated by marked increase in the gingival thickness in the chorion membrane group. The percentage of root coverage, however, was insignificantly more in the control group but the results of the test group at all the three intervals have been very promising.

 The discussion has been written in a very appropriate manner justifying all the significant values that have been addressed in the above tables. The discussion of the findings is suitable, gives answers to the research objectives, and incorporates adequate literature support. The inconsistencies and surprising results are explained in light of previous studies. The distinction between results and inferences is apparent, and the speculations are well supported by the current literature. The statements of various metanalysis and systematic reviews are well incorporated and compared with this study.

 One of the major drawbacks of the study is small sample size which has also been mentioned by the authors. Along with that few other limitations such as the use of microscopes have also been mentioned.

 The conclusions are sound, justified and logically explained, and supported by the results. ****The authors have emphasized the significance of the findings and placed them in the context of past research as well as the work's application.

 The authors have also managed to throw some light on the future prospects.

 This article can be accepted for indexing in its current form. No revisions are required.

Is the work clearly and accurately presented and does it cite the current literature?

Yes

If applicable, is the statistical analysis and its interpretation appropriate?

Yes

Are all the source data underlying the results available to ensure full reproducibility?

Yes

Is the study design appropriate and is the work technically sound?

Yes

Are the conclusions drawn adequately supported by the results?

Yes

Are sufficient details of methods and analysis provided to allow replication by others?

Yes

Reviewer Expertise:

Cancer Genetics and Molecular Medicine.

I confirm that I have read this submission and believe that I have an appropriate level of expertise to confirm that it is of an acceptable scientific standard.
